# Frequency of Online Health Information Seeking and Types of Information Sought Among the General Chinese Population: Cross-sectional Study

**DOI:** 10.2196/30855

**Published:** 2021-12-02

**Authors:** Zihui Xiong, Liang Zhang, Zhong Li, Wanchun Xu, Yan Zhang, Ting Ye

**Affiliations:** 1 School of Medicine and Health Management Tongji Medical College Huazhong University of Science and Technology Wuhan China; 2 Research Center for Rural Health Service Key Research Institute of Humanities & Social Sciences of Hubei Provincial Department of Education Wuhan China; 3 School of Political Science and Public Administration Wuhan University Wuhan China; 4 School of Health Policy and Management Nanjing Medical University Nanjing China

**Keywords:** online health information seeking, sociodemographic factors, information types, Chinese population, information seeking behavior, demography, China, online health information

## Abstract

**Background:**

The internet is one of the most popular health information resources, and the Chinese constitute one-fifth of the online users worldwide. As internet penetration continues to rise, more details on the Chinese population seeking online health information need to be known based on the current literature.

**Objective:**

This study aims to explore the sociodemographic differences in online health information seeking (OHIS), including the frequency of OHIS and the types of online health information sought among the general Chinese population in mainland China.

**Methods:**

A cross-sectional study for assessing the residents’ health care needs with self-administered questionnaires was implemented in 4 counties and districts in China from July 2018 to August 2018. Pearson’s chi-square test was used to identify the sociodemographic differences between infrequent and frequent online health information seekers. We also performed binary logistic regression for the 4 types of online health information as the dependent variables and the sociodemographic factors as the independent variables.

**Results:**

Compared with infrequent online health information seekers, frequent seekers were more likely to be female (infrequent: 1654/3318; 49.85%; frequent: 1015/1831, 55.43%), older (over 60 years old; infrequent: 454/3318, 13.68%; frequent: 282/1831, 15.40%), married (infrequent: 2649/3318, 79.84%; frequent: 1537/1831, 83.94%), and better educated (bachelor’s or above; infrequent: 834/3318, 25.14%; frequent: 566/1831, 30.91%). They were also more likely to earn a higher income (over RMB ¥50k [RMB ¥1=US $0.15641]; infrequent: 1139/3318, 34.33%; frequent: 710/1831, 34.78%), have commercial health insurance (infrequent: 628/3318, 18.93%; frequent: 470/1831, 25.67%), and have reported illness in the past 12 months (infrequent: 659/3318, 19.86%; frequent: 415/1831, 22.67%). Among the 4 health information types, health science popularization was the most searched for information by Chinese online health information seekers (3654/5149, 70.79%), followed by healthy behaviors (3567/5149, 69.28%), traditional Chinese medicine (1931/5149, 37.50%), and medical concerns (1703/5149, 33.07%). The binary logistic regression models showed that males were less likely to seek information on healthy behaviors (adjusted odds ratio [AOR] 0.69, 95% CI 0.61-0.78) and traditional Chinese medicine (AOR 0.64, 95% CI 0.57-0.73), and respondents who had at least 1 chronic disease were more likely to seek information on medical concerns (AOR 1.27, 95% CI 1.07-1.51) and traditional Chinese medicine (AOR 1.26, 95% CI 1.06-1.49).

**Conclusions:**

Sociodemographic factors were associated with the frequency of OHIS and types of information sought among the general Chinese population. The results remind providers of online health information to consider the needs of specific population groups when tailoring and presenting health information to the target population.

## Introduction

Given its rapid development, the internet has become an essential source of health information worldwide. According to Internet World Stats, the number of internet users has reached 5 billion, accounting for more than 60% of the world population [1]. The internet has the highest usage rate among health information sources [2-4]. Although certain groups still rely on traditional media for health information [5], the internet is increasingly growing in popularity as a source of health information.

The advantages of online health information seeking (OHIS) manifest in various aspects. Turning to the internet before seeking a professional diagnosis has the potential to improve the relationship between patients and their physicians, and patients are more inclined to trust in their physicians’ advice by discussing their findings online with these professionals [6-8]. The likelihood of individuals changing their health behavior rises as the frequency of using the internet to retrieve health information increases [9]. For people who live with chronic diseases, the internet may improve their skills in managing their condition [10,11]. However, seeking health information online can be a mixed blessing. Studies have shown that online health information can be inadequate and inaccurate [12-14]. The information may be of questionable quality, leading to hasty, ill-informed, and dangerous health choices [15]. Compared with that available in Western countries, the low-quality online health information seems to be particularly serious in Asian countries [16].

Previous studies have explored how sociodemographic factors influence OHIS behavior. The frequency of OHIS is higher among individuals who are female, younger, better educated, or have a higher household income [9,17,18]. A study that analyzed 2 Pew data sets collected in 2002 and 2012 showed that the sociodemographic factors of gender and education strongly predicted OHIS in both years; meanwhile, age and income were significant predictors in 2012 but not in 2002 [19]. Internet access and OHIS behavior have been shown to vary by race and ethnicity [20-24]. Other factors, such as health status [17] and employment status [24], were also found to be statistically significant in OHIS. A meta-analysis on OHIS revealed that age, gender, education, income, race, and experience were social determinants of OHIS [25]. In summary, most related studies indicate that the crucial characteristics of online health information seekers are age, level of education, income, and ethnicity [26]. Several studies have consistently focused on the predictors of OHIS; however, most of them aimed at identifying determinants that discriminate between online health information seekers and nonseekers [4,20,24,27-29], and elements associated with OHIS among those who have used the internet for health information should also be examined.

Seekers of online health information seek various health topics. Some studies reported information about disease to be the most common topic sought by online health information seekers [19,30]. Other frequently searched topics include symptoms, medication, treatment, exercise and fitness, and nutrition or diet [8,10,30,31]. However, there is no official or agreed upon classification for these common health topics. By reviewing previous studies, these topics can be mainly grouped into 2 types: (1) healthy behaviors, including information related to nutrition or diet, exercise, and body maintenance; (2) and medical concerns, including information related to disease, medications, and treatments [19,32]. Of these 2 types, information on medical concerns is more likely to be sought by people with more health risks [8,31,32]. For instance, compared with the population with no chronic disease, people with chronic conditions are more likely to search about medicines [10]. Overall, evidence shows that medical information seems to be the most popular health information on the internet. However, different population groups have different preferences for health information, and how individuals who seek this information may vary by their characteristics remains obscure.

According to the 47th Statistical Report on China’s internet Development [33], as of December 2020, 9.89 million Chinese had access to the internet, and the penetration rate in China reached 70.4%, a rate higher than the average level in Asia (62.6%) [1]. The Chinese population constitutes one-fifth of the world’s internet users, and easy access to specialized information, such as medical and health care information, accounts for 29.8% of the factors facilitating China’s nonnetizens’ access to the internet [33]. However, academic research on OHIS has mostly collected evidence from Western countries, especially the United States [5,10,21], or has focused on certain groups, such as women [23,34], adolescents [28,35], and patients with chronic disease [10,11]. Studies on the general Chinese population, particularly the Mainland population, are still limited.

According to the literature review, OHIS has not only positive but also negative influences on individuals. With the rise of the internet penetration rate, an increasing number of people will have access to online health information. New health information is difficult to effectively categorize when individuals are overwhelmed by diverse or mixed health information, and an overabundance of health information on the internet can make individuals feel confused and become more anxious about their conditions [36,37]. To ensure that online health information can be effectively used and to understand how individuals can best avoid the negative influence of OHIS, it is crucial to understand the characteristics and preferences of online health information seekers so that practical information can be tailored and presented to the target population. As a result, we aimed to answer 3 questions: (1) Which groups of people are frequent online health information seekers? (2) Which types of online health information do people usually search for? (3) What are the preferences for types of online health information among different population groups? Given that sociodemographic factors have provided an increasingly significant explanation of the variance in OHIS [19], our study aimed to explore sociodemographic differences in OHIS behavior among the general Chinese population, including frequency of OHIS and types of health information sought.

This study used the data of a household survey conducted in 4 counties and districts in Mainland China, which may additionally contribute to the current research. As inaccurate information and the inability to find information may discourage people from retrieving professional information [38], our findings may offer additional knowledge to online health information providers so that appropriate health information content can be tailored and presented. Therefore, we believe this study can significantly contribute to online health information management and promotion in China.

## Methods

### Study Design

From July 2018 to August 2018, the Research Center for Rural Health Services carried out a study to assess residents’ health care needs. Based on the assessment of socioeconomic development and geographic distribution and suggestions from experts [39], 4 counties and districts across China were purposely selected as the survey areas, including 2 districts in urban China (Xiling in central China, Futian in eastern China) and 2 counties in rural China (Dangyang in central China, Sinan in western China) [40]. 

A multistage stratified random sampling was implemented to select representative samples of the districts’ and counties’ populations. In the sample size calculation, the design effect was set at 2.5, and the allowable error at the significance level of .05; the chronic disease prevalence was 21.338% according to the 2013 National Health Service Survey [41], and thus the minimum sample size in each survey area was calculated to be 3584 individuals. Given that a family in China has an average of 2.9 members [41], at least 1235 families in every study site needed to be investigated. In the 2 counties (Dangyang and Sinan), 5 towns in each county were randomly selected according to the distance to the county hospitals. Meanwhile, 6 villages in each town were randomly selected according to the distance to the township hospitals. In the 2 districts (Xiling and Futian), 5 streets in each district were randomly selected according to the distance to the medical centers. To ensure that at least 248 families in each town or street were effectively interviewed, 1 other town or street in each county or district was selected as a backup in case of refusal, closed household, or removal of defective questionnaires. We resampled and surveyed until the final sample size met the estimated requirement. The surveyed families were systematically sampled in the rosters of residents from the village or street councils. Under the guidance of well-trained interviewers, face-to-face interviews were conducted, and self-administrated questionnaires were used on all the members of the sampled families. Finally, data from 15,126 respondents in 5547 families were collected in total: 3310 individuals in 1360 families in 30 villages in Dangyang, 3983 individuals in 1355 families in 30 villages in Sinan, 4386 individuals in 1513 families in 6 streets in Futian, and 3447 individuals in 1319 families in 5 streets in Xiling. A total of 12,646 individuals above 15 years of age needed to answer the questions related to OHIS, and 5354 respondents (5354/12,646, 42.34%) reported using the internet to seek health information. Among the online health information seekers, those who failed to provide information for all variables queried in this study were excluded, and 5149 individuals were finally included in the analysis.

Ethical approval was obtained from the Ethics Committee of Tongji Medical College, Huazhong University of Science and Technology (#IORG0003571).

### Variables

#### Sociodemographic Factors

The survey questionnaires were divided into several parts, including basic family information, individual information, health status, health care use, and health promotion need. Sociodemographic factors, including gender, age, marital status, area of residence, education level, annual household income per capita, employment, health insurance, having at least 1 chronic disease, and reported illness in the past 12 months, were considered to be independent variables. The coding of independent variables is shown in Table 1.

**Table 1:** Coding of independent variables.

Variables	Assignment
Gender	Female=0; male=1
Age (years)	15-45=1; 46-60=2; >60=3
Marital status^a^	Single=0; married=1
Area	Rural=0; urban=1
Education level	Junior high school or below=1; senior high school =2; 3-year college=3; bachelor’s or above=4
Annual household income per capita (RMB ¥^b^)	<10k=1; 10k-30k=2; 30k-50k=3; >50k=4
Employment^c^	Unemployed=0; employed=1
Types of primary health insurance	Urban employee basic medical insurance=1; urban-rural resident basic medical insurance=2; others^d^=3
Having commercial health insurance	No=0; yes=1
Having at least 1 chronic disease	No=0; yes=1
Reported illness in the past 12 months	No=0; yes=1

^a^Marital status was divided into 2 categories: single (unmarried, divorced, or widowed) and married (married).

^b^RMB ¥1=US $0.15641.

^c^Employment was recoded as 2 groups: unemployed (unemployed, students, and retired) and employed (employed).

^d^Others included no primary health insurance and health insurance outside mainland China.

#### Frequency of OHIS

Most frequency response scales use subjective terms as scale labels, such as the 3-point frequency scale (usually, sometimes or partially, never) and 4-point frequency scale (never, seldom/hardly ever, sometimes, often) [9,42,43]. In this study, the measurement of the frequency of OHIS was adapted from previous studies, which were based on respondents’ subjective ratings. Respondents were asked to rate the frequency of OHIS on a 3-point frequency scale (frequent, infrequent, never), and those who selected “Frequent” and “Infrequent” were classified as frequent and infrequent online health information seekers, respectively. Given that our study participants were those who had used online health information, respondents who selected “Never” were excluded.

#### Types of Online Health Information

In the original survey, respondents were asked whether they had searched for the following health-related contents: sports and fitness, health science popularization, traditional Chinese medicine (TCM), women’s health, nutrition and diet, medication guidance, disease consultation, health status monitoring, and others. In this study, 6 items were selected and then grouped into 4 types to be analyzed: (1) healthy behaviors, including sport and fitness, and nutrition and diet; (2) medical concerns, including medication guidance and disease consulting; (3) TCM, which may particularly be sought by Chinese users [31,44]; and (4) health science popularization, an emerging type of health information that aims at improving people’s health literacy, which the National Health Commission of China has strongly supported in recent years. According to the National Health Commission of China, information on health science popularization mainly includes scientific knowledge about health technologies, ideas, methods, and skills, which is particularly disseminated in a way that is easy to understand, accept, and participate in [45]. Of the 4 types of online health information, the first 2 contain the common health topics frequently sought by the general population worldwide, and the analysis of the latter 2 may be helpful to better understanding the OHIS behavior of the general population in the setting of Mainland China. Each type as a dependent variable was dichotomized (yes or no).

### Statistical Analysis

First, Pearson’s chi-square test was used to compare sociodemographic differences between infrequent and frequent online health information seekers. Second, descriptive analysis was applied to show the prevalence of different types of online health information. Finally, binary logistic regression was conducted on each type of online health information to identify the sociodemographic differences in searches for health information types, and the adjusted odds ratio (AOR) and 95% CI were reported. The significance level was set at a *P* value of .05.

## Results

### Sociodemographic Information of the Respondents

Table 2 shows an overview of the main sociodemographic characteristics of the study population and differences in the frequency of OHIS. Online health information seekers comprised more females (2669/5149, 51.84), and most of them lived in urban China (3993/5149, 77.55%). Almost two-thirds (3318/5149, 64.44%) of respondents reported infrequently using the internet to seek health information. Among people who were frequent seekers of online health information, more than half (1015/1831, 55.43%) were female. The comparison between the 2 groups revealed that differences in terms of gender (*P*<.001), age (*P*=.01), marital status (*P*<.001), education level (*P*<.001), annual household income per capita (*P*<.001), possession of commercial insurance (*P*<.001), and reported illness in the past 12 months (*P*=.02) were statistically significant. In contrast, area (*P*=.12), employment (*P*=.79), types of primary health insurance (*P*=.57), and having at least 1 chronic disease (*P*=.14) were not significantly different. Compared with infrequent online health information seekers, frequent seekers were more likely to be females (infrequent: 1654/3318, 49.85%; frequent: 1015/1831, 55.43%), were older (over 60 years old; infrequent: 454/3318, 13.68%; frequent: 282/1831, 15.40%), were more likely to be married (infrequent: 2649/3318, 79.84%; frequent: 1537/1831, 83.94%), were better educated (bachelor’s or above; infrequent: 834/3318, 25.14%; frequent: 566/1831, 30.91%), earned a higher income (over RMB ¥50k [RMB ¥1=US $0.15641; frequent: 1139/3318, 34.33%; frequent: 710/1831, 34.78%), were more likely to have commercial health insurance (infrequent: 628/3318, 18.93%; frequent: 470/1831, 25.67%), and were more likely to have reported illness in the past 12 months (infrequent: 659/3318, 19.86%; frequent: 415/1831, 22.67%).

**Table 2:** Sociodemographic characteristics of the respondents by frequency of OHIS (online health information seeking).

Sociodemographic characteristics		Overall (N=5149), n (%)	Infrequent (N=3318), n (%)	Frequent (N=1831), n (%)	*P* value	
**Gender**						<.001
	Male	2480 (48.16)	1664 (50.15)	816 (44.57)		
	Female	2669 (51.84)	1654 (49.85)	1015 (55.43)		
**Age**						.01
	15-45 years	3015 (58.56)	1992 (60.04)	1023 (55.87)		
	46-60 years	1398 (27.15)	872 (26.28)	526 (28.73)		
	>60 years	736 (14.29)	454 (13.68)	282 (15.40)		
**Marital status**						<.001
	Single	963 (18.70)	669 (20.16)	294 (16.06)		
	Married	4186 (81.30)	2649 (79.84)	1537 (83.94)		
**Area**						0.12
	Rural	1156 (22.45)	767 (23.12)	389 (21.25)		
	Urban	3993 (77.55)	2551 (76.88)	1442 (78.75)		
**Education level**						<.001
	Junior high school or below	1476 (28.67)	1029 (31.01)	447 (24.41)		
	Senior high school	1547 (30.04)	996 (30.02)	551 (30.09)		
	3-year college	726 (14.10)	459 (13.83)	267 (14.58)		
	Bachelor’s or above	1400 (27.19)	834 (25.14)	566 (30.91)		
**Annual household income per capita^a^**						<.001
	<10k	578 (11.23)	406 (12.24)	172 (9.39)		
	10k-30k	1600 (31.07)	1071 (32.28)	529 (28.89)		
	30k-50k	1122 (21.79)	702 (21.16)	420 (22.94)		
	>50k	1849 (35.91)	1139 (34.33)	710 (38.78)		
**Employment**						.79
	Unemployed	1764 (34.26)	1141 (34.39)	623 (34.03)		
	Employed	3385 (65.74)	2177 (65.61)	1208 (65.97)		
**Types of primary health insurance**						.57
	UEBMI^b^	2331 (45.27)	1494 (45.03)	837 (45.71)		
	URBMI^c^	2620 (50.88)	1702 (51.30)	918 (50.14)		
	Other	198 (3.85)	122 (3.68)	76 (4.15)		
**Having commercial health insurance**						<.001
	No	4051 (78.68)	2690 (81.07)	1361 (74.33)		
	Yes	1098 (21.32)	628 (18.93)	470 (25.67)		
**Having at least 1 chronic disease**						.14
	No	4154 (80.68)	2697 (66.40)	1457 (79.57)		
	Yes	995 (19.32)	621 (57.19)	374 (20.43)		
**Reported illness in the past 12 months**						.02
	No	4075 (79.14)	2659 (80.14)	1416 (77.33)		
	Yes	1074 (20.86)	659 (19.86)	415 (22.67)		

^a^Income is reported in renminbi ¥. RMB ¥1=US $0.15641.

^b^UEBMI: urban employee basic medical insurance.

^c^URBMI: urban-rural resident basic medical insurance.

### Types of Online Health Information Searched

Respondents reported 1 or more different types of health information they sought online. A majority of the respondents (3654/5149, 70.79%) had used the internet to seek health science popularization information, which was followed in popularity by healthy behaviors (3567/5149, 69.28%), TCM, and medical concerns (1703/5149, 33.07%). The prevalence of the searches for the 4 types of online health information is shown in Table 3.

**Table 3:** Prevalence of types of online health information sought by the study participants.

Types of online health information	Prevalence (N=5149), n (%)
Healthy behaviors	3567 (69.28)
Medical concerns	1703 (33.07)
Traditional Chinese medicine	1931 (37.50)
Health science popularization	3645 (70.79)

### Sociodemographic Factors Associated With the Types of OHIS

The results of the binary logistic regression models illustrated sociodemographic differences in seeking the 4 types of online health information (Table 4). Being male was significantly associated with a lower possibility of seeking healthy behaviors (AOR 0.69, 95% CI 0.61-0.78) and TCM (AOR 0.64, 95% CI 0.57-0.73). Compared with individuals aged between 15 and 45 years, middle-aged individuals were more likely to seek TCM (AOR 1.86, 95% CI 1.61-2.15) and health science popularization (AOR 1.20, 95% CI 1.02-1.40), while older adults were more likely to seek information for medical concerns (AOR 1.40, 95% CI 1.12-1.76) and TCM (AOR 2.16, 95% CI 1.72-2.70). Compared with single respondents, married respondents were more likely to seek TCM (AOR 1.22, 95% CI 1.03-1.44) and health science popularization (AOR 1.20, 95% CI 1.02-1.41) but were less likely to seek healthy behaviors (AOR 0.67, 95% CI 0.56-0.80). Living in urban China was associated with increased odds of seeking TCM (AOR 1.23, 95% CI 1.01-1.52). Higher education attainment was correlated with a higher possibility of seeking healthy behaviors (3-year college: AOR 1.56, 95% CI 1.26-1.95; bachelor’s or above: AOR 1.33, 95% CI 1.10-1.62), medical concerns (senior high school: AOR 1.19, 95% CI 1.01-1.39; bachelor’s or above: AOR 1.46, 95% CI 1.20-1.77), and health science popularization (bachelor’s or above: AOR 1.50, 95% CI 1.23-1.83). Lower annual household income per capita was associated with increased odds of seeking healthy behaviors (RMB ¥10k-30: AOR 1.21, 95% CI 1.02-1.44) but decreased odds of seeking TCM (<RMB ¥10k: AOR 0.70, 95% CI 0.54-0.91; RMB ¥10k-30k: AOR 0.76, 95% CI 0.64-0.89). Respondents with urban-rural resident basic medical insurance were less likely to seek health science popularization than those with urban employee basic medical insurance (AOR 0.86, 95% CI 0.74-1.00). Having commercial health insurance was associated with a higher possibility to seek healthy behaviors (AOR 1.55, 95% CI 1.32-1.83) and TCM (AOR 1.27, 95% CI 1.10-1.47) but a lower possibility to seek medical concerns (AOR 0.82, 95% CI 0.70-0.95) and health science popularization (AOR 0.83, 95% CI 0.71-0.96). Individuals who had at least 1 chronic disease were more likely to seek medical concerns (AOR 1.27, 95% CI 1.07-1.51) and TCM (AOR 1.26, 95% CI 1.06-1.49). Similarly, those who reported illness during the past 12 months were also more likely to seek medical concerns (AOR 1.41, 95% CI 1.21-1.65) and TCM (AOR 1.17, 95% CI 1.00-1.38).

**Table 4:** Binary logistic regression models for AORs and 95% CIs of reporting the search for each type of online health information.

Sociodemographic Characteristics			Healthy behaviors, AOR^a^ (95% CI)		Medical concerns, AOR (95% CI)		Traditional Chinese medicine, AOR (95% CI)		Health science popularization, AOR (95% CI)
**Gender**									
	Female (Ref^b^)								
	Male	0.69 (0.61-0.78)**		1.11 (0.98-1.25)		0.64 (0.57-0.73)**		1.13 (1.00-1.28)	
**Age**									
	15-45 years (Ref)								
	46-60 years	1.07 (0.92-1.25)		0.93 (0.80-1.08)		1.86 (1.61-2.15)**		1.20 (1.02-1.40)*	
	>60 years	0.85 (0.67-1.07)		1.40 (1.12-1.76)**		2.16 (1.72-2.70)**		1.14 (0.90-1.46)	
**Marital status**									
	Single (Ref)								
	Married	0.67 (0.56-0.80)**		1.17 (0.99-1.37)		1.22 (1.03-1.44)*		1.20 (1.02-1.41)*	
**Area**									
	Rural (Ref)								
	Urban	0.97 (0.79-1.19)		0.95 (0.77-1.16)		1.23 (1.01-1.52)*		1.05 (0.85-1.29)	
**Education level**									
	Junior high school or below (Ref)								
	Senior high school	1.16 (0.99-1.37)		1.19 (1.01-1.40)*		1.04 (0.89-1.22)		1.14 (0.97-1.34)	
	3-year college	1.56 (1.26-1.95)**		1.21 (0.98-1.50)		1.05 (0.85-1.29)		1.19 (0.96-1.48)	
	Bachelor’s or above	1.33 (1.10-1.62)**		1.46 (1.20-1.77)**		0.89 (0.74-1.08)		1.50 (1.23-1.83)**	
**Annual household income per capita^c^**									
	>50k (Ref)								
	<10k	0.94 (0.72-1.21)		0.84 (0.65-1.09)		0.70 (0.54-0.91)**		0.99 (0.76-1.28)	
	10k-30k	1.21 (1.02-1.44)*		1.03 (0.87-1.22)		0.76 (0.64-0.89)**		1.11 (0.94-1.32)	
	30k-50k	1.17 (0.98-1.38)		1.09 (0.92-1.28)		1.01 (0.86-1.19)		1.19 (1.00-1.41)	
**Employment**									
	Unemployed (Ref)								
	Employed	0.88 (0.75-1.04)		1.10 (0.94-1.29)		1.12 (0.96-1.31)		0.92 (0.78-1.07)	
**Types of primary health insurance**									
	Urban employee basic medical insurance (Ref)								
	URBMI^d^	0.96 (0.83-1.11)		1.05 (0.91-1.21)		1.06 (0.92-1.22)		0.86 (0.74-1.00)*	
	Other	0.93 (0.67-1.29)		1.12 (0.81-1.53)		0.79 (0.58-1.09)		0.69 (0.50-0.94)*	
**Having commercial health insurance**									
	No (Ref)								
	Yes	1.55 (1.32-1.83)**		0.82 (0.70-0.95)*		1.27 (1.10-1.47)**		0.83 (0.71-0.96)*	
**Having at least 1 chronic disease**									
	No (Ref)								
	Yes	1.02 (0.85-1.22)		1.27 (1.07-1.51)*		1.26 (1.06-1.49)**		0.96 (0.80-1.15)	
**Reported illness during the past 12 months**									
	No (Ref)								
	Yes	0.88 (0.75-1.03)		1.41 (1.21-1.65)**		1.17 (1.00-1.38)*		1.09 (0.93-1.29)	

^a^AOR: adjusted odds ratio.

^b^Ref: reference group.

^c^Income is reported in renminbi ¥. RMB ¥1=US $0.15641.

^d^URBMI: urban-rural resident basic medical insurance.

*P<.05; **P<.01.

## Discussion

### Principal Findings and Comparison With Prior Work

In this population-based study, we examined the sociodemographic differences in frequency of OHIS and types of online health information sought among the general population in China. This study corroborated the findings of a great deal of previous work in that individuals who were female, better educated, and had a higher household income were positively associated with OHIS [17,18,20,23]. The differences in frequency of OHIS among socioeconomically defined groups implied that inequality in health communication might also exist in China, which was consistent with findings of a previous study from Hong Kong [29]. We also found that living areas had no significant difference between infrequent and frequent online health information seekers, while this difference might exist between seekers and nonseekers [20]. In addition, the sociodemographic characteristics included in the analysis were not consistently associated with the types of online health information sought among the Chinese population.

One unanticipated finding is that respondents who frequently sought online health information were more likely to be middle-aged or older adults. This result deviated from the bulk of previous studies, which showed that younger age groups preferred the internet as a source of health information [17,20,23]. Regardless of sources, however, individuals with older age are more likely to search for health information [4,29]. Moreover, a study from Hong Kong found that older adults were more frequently exposed to health information from instant messaging such as WeChat [42]. Given that our study participants were those who had already searched on the internet for health information, a possible reason for this discrepancy is that once older adults have obtained access to online health information, they are likely to seek it more frequently than are younger individuals, thus suggesting the strong demand for health information among middle-aged or older adults in China. However, additional research effort is needed to verify this hypothesis further. As the population ages in China, the health department of the government and other health organizations should take the internet into account as an effective resource to intervene with the older adults’ health behaviors. Given that digital health is not reaching all seniors equally and that this disparity probably results in differences in health outcomes [23], the skills and knowledge of older adults should be reinforced so that more of them can use and benefit from online health information. We also found that the married group had a higher proportion of frequent seekers of online health information, suggesting that married respondents might also seek health information for their family members. A study among German older adults found that internet users were more likely to be married or with a partner [46]. Another study revealed that middle-aged adults were most likely to use the internet to search for information about a condition of a loved one [47]. The association between marital status and the frequency of OHIS may coincide with the association between age and the frequency of OHIS, which may also mean that older people seek out health information for themselves as well as for their partners [47]. Therefore, meeting the health information needs of older adults (eg, information on medical concerns and TCM) is beneficial not only for themselves but also for their partners.

Health science popularization was the most searched for type of online health information among Chinese respondents, and medical concerns was the least searched for type of information. Conversely, earlier literature mostly reported that medical information was the most searched for type of online information [19,30]. This discrepancy may have several causes. On the one hand, the cultural differences between China and other countries, especially Western countries, might have played a role. In recent years, the National Health Commission of China has encouraged the dissemination of health science popularization information in various ways [45]. The increasing popularity of social media and the ever-growing number of official accounts of health science popularization might have attracted many Chinese netizens to follow such information. For instance, by comparing Americans and Hong Kongers, one study revealed that the latter were more likely to trust and use information from social websites [48]. On the other hand, only a minority of respondents in our study had a chronic disease (995/5149, 19.32%) or reported illness in the past 12 months (1074/5149, 20.86%), but they were found more likely to seek information about medical concerns. In addition, the results also showed that respondents over 60 years of age were more likely to seek information about medical concerns, but only 14.29% (736/5149) of the study participants were over 60 years old.

Consistent with previous studies, our study confirmed that people facing health challenges preferred medical information rather than information on healthy behaviors [10,32], which indicated that individuals with more health risks were more eager to improve their current health status. Therefore, people’s preferences for health information may be influenced by distinct motivations [49]. More research needs to be conducted to understand Chinese people’s motivation in searching for OHIS. Information on healthy behaviors was found to be more likely to be sought by female or better-educated respondents, which was broadly in line with previous studies [50-52]. Our study also found that single respondents were more likely to seek information on healthy behaviors. The sociodemographic differences in preferences for online health information highlight the importance of distinguishing online health information seekers when tailoring and presenting needed information to targeted users.

Information on TCM was more likely to attract attention from older adults, especially older women with chronic diseases. A cross-sectional study focused on older adults in Shanghai indicated that females were more likely to trust and use TCM [53]. Another study showed that having chronic lung disease was significantly associated with higher TCM practitioner utilization [54]. Indeed, having a chronic condition was identified as one of the main factors that induced people to use TCM in China [55]. As the significant factors found in our study were consistent with those in several previous studies, the preference for online health information about TCM might partly reflect the preference for the use of TCM. Our findings also showed that 37.5% of the respondents had sought TCM information, while a study investigating OHIS behavior of Chinese college students reported that only 15.6% sought TCM information online [44]. Given that TCM is widely accepted and used among the middle-aged and older Chinese population [55], along with the advantages of online health information mentioned before, we believe the provision of TCM-related information on the internet may promote the use of TCM and improve the management of chronic disease in China.

### Limitations

Our study has several limitations. First, the content about the motivation for OHIS (eg, for self-care, treating families, and improving lifestyles) and health information sources on the internet (eg, social media and professional websites) were not surveyed, thus resulting in fewer details on the OHIS behavior of Chinese internet users. Second, differences in online health information type classification made our results difficult to compare with those of previous studies. Finally, the research from which this study originated was implemented in 2018. The degree and diversity of internet use has grown since the beginning of the COVID-19 epidemic in 2020. As internet hospitals [56,57], health quick response codes, and antiepidemic science popularization online have been developed and used during the pandemic, the penetration rate of online health information users, especially online medical service users, continues to increase [33]. In addition, the status quo of OHIS in China needs to be further investigated in future research.

### Conclusions

This study enriches the current research on the OHIS behavior of the Chinese population. Furthermore, it presents additional knowledge that providers of online health information, government health departments, and other organizations can use. The significant factors found in our study highlight the necessity of identifying the characteristics of the typical online health information users in China, especially age, gender, education level, marital status, and health status, when targeting and developing health interventions by offering health information on the internet. Those who provide health information online should be aware of the needs of specific population groups so that targeted strategies to promote health are presented appropriately.

## Abbreviations

AOR: adjusted odds ratio
OHIS: online health information seeking
TCM: traditional Chinese medicine
